# Serotonin Syndrome Following Low-Dose Sertraline: A Case Report

**DOI:** 10.1192/j.eurpsy.2024.1175

**Published:** 2024-08-27

**Authors:** C. Y. Chan

**Affiliations:** general psychiatry, institute of mental health, singapore, Singapore

## Abstract

**Introduction:**

Serotonin syndrome is a potentially life-threatening condition that is precipitated by the use of serotonergic medications, Selective Serotonin Reuptake Inhibitors (SSRIs) and Monoamine Oxidase Inhibitors (MAOIs). It usually occurs when high doses of serotonergic drugs are prescribed. It is a medical emergency that requires prompt recognition, cessation of offending drugs and supportive therapy.

**Objectives:**

We present a case of serotonin syndrome that occurred in a patient who was prescribed a low dose of sertraline, and aim to highlight the importance of early detection of this severe condition.

**Methods:**

Details of the case were described. Information was gathered based on medical records.

**Results:**

Patient M was a 29-year-old Malay male with a history of major depressive disorder, who was previously trialed on fluvoxamine 100mg every night but subsequently switched to and maintained on sertraline 75mg every night in 2020. He then defaulted follow up appointments. In 2023, he presented to the emergency services for a suicide attempt and was diagnosed with major depressive disorder with psychotic features. He was restarted on sertraline 50mg every night and risperidone 0.5mg every night was newly started. Two days later, sertraline was increased to 100mg every night. Two days following this increase, he was noted to have altered mental state, fever of 39.3-degree celcius, tachycardia of 120 beats per minute, ocular clonus and generalized hyperreflexia. Sertraline and risperidone were immediately stopped. Blood tests including creatine kinase, lumbar puncture and magnetic resonance imaging (MRI) of the brain did not show any abnormalities. After stopping of the medications, the patient’s symptoms resolved within 24 hours. Based on clinical symptoms and a normal creatine kinase level, neuroleptic malignant syndrome (NMS) was ruled out. Subsequently, he was restarted on risperidone 0.5mg and mirtazapine 7.5mg every night. He developed symptoms of serotonin syndrome with a low dose of sertraline. Symptoms resolved after the discontinuation of the SSRI.

**Conclusions:**

In this case, differential diagnoses of serotonin syndrome were also considered, such as NMS, encephalitis, meningitis and thyroid storm. NMS was less likely due to the rapid onset of onset and resolution of symptoms. Encephalitis and meningitis were unlikely in view of normal MRI brain and lumbar puncture findings.

There have been case reports of serotonin syndrome developing with lower doses of an SSRI in the pediatric population. There is, however, a lack of literature describing serotonin syndrome with low doses of SSRI in the adult population. To avoid a missed diagnosis, clinicians should monitor closely for SSRI toxicity, including serotonin syndrome, even when low doses of serotonergic drugs are used.

**Disclosure of Interest:**

None Declared

